# BEL/Pao retrotransposons in metazoan genomes

**DOI:** 10.1186/1471-2148-11-154

**Published:** 2011-06-04

**Authors:** Nicole de la Chaux, Andreas Wagner

**Affiliations:** 1 Department of Biochemistry, University of Zurich, Winterthurerstrasse 190, 8057 Zurich, Switzerland; 2 The Swiss Institute of Bioinformatics, Basel, Switzerland; 3 The Santa Fe Institute, Santa Fe, NM, USA

## Abstract

**Background:**

Long terminal repeat (LTR) retrotransposons are a widespread kind of transposable element present in eukaryotic genomes. They are a major factor in genome evolution due to their ability to create large scale mutations and genome rearrangements. Compared to other transposable elements, little attention has been paid to elements belonging to the metazoan BEL/Pao subclass of LTR retrotransposons. No comprehensive characterization of these elements is available so far. The aim of this study was to describe all BEL/Pao elements in a set of 62 sequenced metazoan genomes, and to analyze their phylogenetic relationship.

**Results:**

We identified a total of 7,861 BEL/Pao elements in 53 of our 62 study genomes. We identified BEL/Pao elements in 20 genomes where such elements had not been found so far. Our analysis shows that BEL/Pao elements are the second-most abundant class of LTR retrotransposons in the genomes we study, more abundant than Ty1/Copia elements, and second only to Ty3/Gypsy elements. They occur in multiple phyla, including basal metazoan phyla, suggesting that BEL/Pao elements arose early in animal evolution. We confirm findings from previous studies that BEL/Pao elements do not occur in mammals. The elements we found can be grouped into more than 1725 families, 1623 of which are new, previously unknown families. These families fall into seven superfamilies, only five of which have been characterized so far. One new superfamily is a major subdivision of the Pao superfamily which we propose to call Dan, because it is restricted to the genome of the zebrafish *Danio rerio*. The other new superfamily comprises 83 elements and is restricted to lower aquatic eumetazoans. We propose to call this superfamily Flow. BEL/Pao elements do not show any signs of recent horizontal gene transfer between distantly related species.

**Conclusions:**

In sum, our analysis identifies thousands of new BEL/Pao elements and provides new insights into their distribution, abundance, and evolution.

## Background

Transposable elements (TEs) are DNA sequences that have the ability to replicate within a genome using a variety of mechanisms [1]. They are present in almost all eukaryotic genomes, and they play an important role in genome evolution by creating genetic variation through their mobility [2]. Although most new TE insertions have a negative effect on the host's fitness, they significantly contribute to genome evolution [3]. TEs can be divided into two classes based on their replication mechanism: retrotransposons (class I) and DNA transposons (class II) [1]. While retrotransposons use an RNA intermediate for transposition, DNA transposons use a DNA intermediate. Because of their replication mechanism retrotransposons are generally present in larger numbers than DNA transposons, and can reach very high copy numbers. They also show a broader phylogenetic distribution [4]. Retrotransposons can be further subdivided into two major classes based on whether they have long terminal repeat (LTR) sequences, i.e., LTR retrotransposons and non-LTR retrotransposons [4]. We here do not discuss non-LTR retrotransposons further.

LTR retrotransposons are similar to retroviruses [5] and consist of a protein-coding region which is flanked by two LTR sequences. The LTR sequences regulate transcription and play important roles in the copying of the element's RNA into DNA. The protein coding region usually contains one or two open reading frames (ORFs) which are similar to *gag *and *pol *genes from retroviruses. While the *gag *gene encodes proteins that form virus-like particles, the *pol *gene encodes various enzymatic activities like aspartic protease, reverse transcriptase, and integrase. A small fraction of LTR elements additionally contains an *env *gene captured from retroviruses [6], where this gene is important for the infectivity of the virus. In the few LTR elements where this gene occurs, it is usually non-functional [7-9].

LTR retrotransposons can be further divided into four subclasses based on their sequence similarity and various structural features: Ty1/Copia, Ty3/Gypsy, BEL/Pao (also sometimes named only BEL or Pao) and DIRS [10]. Most of the known LTR elements belong to the two large Ty1/Copia and Ty3/Gypsy subclasses. Elements from these subclasses are present in almost all eukaryotic genomes and are already intensely studied. In contrast, the other two subclasses contain fewer elements and were identified only recently [11,12]. While elements from the Ty1/Copia, Ty3/Gypsy and DIRS subclasses are widespread in eukaryotic genomes, BEL/Pao elements are only present in metazoan genomes, suggesting that they arose later in eukaryote evolution or that they have been lost (or not yet identified) in several major eukaryotic lineages. Even in animal genomes they do not show a continuous distribution. For example, no elements have been identified in mammals so far.

The evolutionary history of the BEL/Pao subclass is not well understood. More and more BEL/Pao elements are being reported in different genomes, which raises the question if the BEL/Pao subclass is really as small as previously assumed [13-17]. Around 160 different BEL/Pao families in approximately 40 species have been reported and the number is still growing [6,11,14,16,18-21]. BEL/Pao elements are usually between 4.2-10 kb long. They are flanked by LTR sequences that are 0.2-1.2 kb long [22]. BEL/Pao elements show the same domain arrangement as elements of the Ty3/Gypsy subclass and few BEL/Pao elements contain an *env *gene [22].

Contradictory evolutionary relationships for elements within the BEL/Pao subclass and for the relationship of the BEL/Pao subclass to the other three subclasses have been reported [6,16,23]. Current research focuses mainly on the identification of new BEL/Pao elements in *one *genome of interest. For studying the evolutionary history of these elements, just a few elements from other organisms are typically added as outgroups [16,24]. The most comprehensive phylogenetic analysis thus far was carried out by Copeland et al. (2005), who analyzed the phylogenetic relationship of 20 different BEL/Pao elements. Although these authors identified five distinct BEL/Pao lineages in insects, nematodes and vertebrates, namely Tas, BEL, Pao, Sinbad, and Suzu [16], no exhaustive analysis of the BEL/Pao subclass of LTR elements is available. Here, we search for BEL/Pao elements in 62 non-mammalian metazoan and in 11 mammalian genomes. We use a de novo search approach to identify all BEL/Pao elements in this set of genomes. After separating the elements into families, we study the phylogenetic relationship between these families, and extend the current classification of BEL/Pao elements. An origin in very early metazoan evolution is often assumed for BEL/Pao elements, but no conclusive evidence exists. Alternatively, BEL/Pao elements might have evolved later, only in a subset of metazoan genomes, and were then transmitted to other metazoan phyla by horizontal transfer. This might explain why, for example, no BEL/Pao elements are present in mammalian genomes. Our sequence data allows us to address this possibility in a preliminary fashion.

## Results

### Element occurences

**Figure 1:**
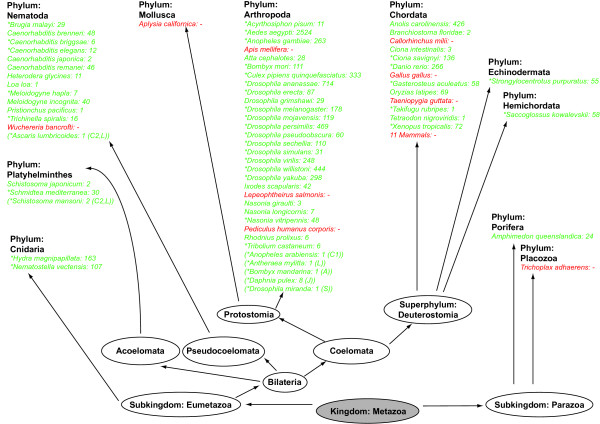
**Overview over analyzed genome sequences and their taxonomic classification**. The names of 62 non-mammalian species whose genomes we analyzed are grouped by phylum. 11 additional mammalian genomes we analyzed are summarized as "11 Mammals". Next to each species, the number of BEL/Pao elements we identified is shown. If we were not able to identify any element in one genome, the genome name is shown in red. Genome sequences where BEL/Pao elements had been already identified previously are marked with an asterix (*). For completeness we list seven additional species where no complete genome sequence was available but where BEL/Pao elements had been identified previously (shown in parentheses). A: Abe et al. (2001) [20], C1: Cook et al. (2000) [13], C2: Copeland et al. (2005) [16], J: Jurka and Kohany (2010) [21], L: Llorens et al. (2008) [23], S: Steinemann and Steinemann (1997) [19].

Figure 1 shows the species in whose genomes we searched for BEL/Pao elements. Species in which our approach did identify BEL/Pao elements are highlighted in green. Species without BEL/Pao elements are shown in red. The taxonomic range of the species we analyzed is broad. It includes both eumetazoa and parazoa, bilateria and radiata, protostomes and deuterostomes, and comprises species from 11 different phyla. Of the 62 genomes we analyzed 27 (44 percent) are from arthropods, reflecting a bias in currently available genome sequences.

The majority of species whose genomes we analyzed belong to one of the three eumetazoan phyla Nematoda, Arthropoda and Chordata. In all of these three phyla BEL/Pao elements had been identified previously. In contrast to previous work, we also had access to species from new superphyla and from the subkingdom parazoa. This allowed us to identify elements in these new metazoan clades as well. For example, no BEL/Pao element has been identified thus far in the subkingdom parazoa. We identified 24 BEL/Pao elements in the sponge *Amphimedon queenslandica*, a member of this subkingdom. In addition, we identified new BEL/Pao elements in the cnidarian, echinoderm, and hemichordate phyla.

In total, we identified 7,861 BEL/Pao elements in 53 of our 62 genomes, including 20 species where no BEL/Pao elements have been identified before. These elements include full length elements and fragments with a minimum length of 2,000 base pairs. Previous studies reported around 160 BEL/Pao families, but in most genomes the copy number of these families has not been reported [6,11,14,16,18,25]. Our analysis thus is the first to determine the abundance of BEL/Pao elements in multiple genomes. The nucleotide sequences of all BEL/Pao elements are listed in additional file 1.

### Variable abundance of elements

The abundance of BEL/Pao elements is highly variable between different species. Nine of our 62 studied genomes do not contain any BEL/Pao elements, and 14 further species contain no more than ten elements. The relative majority of species (22 in total) contain between eleven and 100 elements, and 16 species contain between 101 and 1000 elements. Only one species, the yellow fever mosquito *Aedes aegypti*, harbors more than 1000 elements (a total of 2524 elements). The number of BEL/Pao elements in each species' genome is listed in additional file 2 and in Figure 1. Previous results reported that no BEL/Pao elements are present in mammalian genomes [16]. We could confirm this finding using eleven mammalian genomes which we analyzed in addition to the 62 genomes we just discussed (see Methods for list of genome names).

Because genome size can influence the abundance of elements in a species [26], we compared the number of BEL/Pao elements per mega base pair (Mbp) between species. Additional file 3A shows a histogram of this LTR element density for the species we studied. The number of BEL/Pao copies ranges from less than 0.01 copies per Mbp in 17 species to 3.55 copies per Mbp in the fruit fly *Drosophila ananassae*. Most genomes contain fewer than one BEL/Pao element per Mbp. Only the mosquito and fruit fly genomes contain more. Five species have between one to two copies per Mbp. These are (in ascending order of element density) the mosquito *Anopheles gambiae *(mean copy number per Mbp 1.14), the fruit flies *Drosophila virilis *(1.34), *Drosophila melanogaster *(1.37), the mosquito *Aedes aegypti *(1.85), and the fruit fly *Drosophila yakuba *(1.96). Two fruit fly species have between two and three copies per Mbps (*D. willistoni *(2.14) and *D. persimilis *(2.9)). The species with the highest overall copy number (*A. aegypti*) is not the species with the highest density of BEL/Pao elements. This observation can be explained by the fact that *A. aegypti *has, with 1.3 Gb, a more than 6 times larger genome than the mosquito and fruit fly genomes [26]. Additional file 3B shows that the total copy number per species and the number of copies per Mbps show a statistically significant association for the genomes we analyzed (Spearman's rank correlation coefficient 0.78, *p *= 5 × 10 ^-12^, n = 53).

### BEL/Pao elements are the second most abundant subclass of LTR retrotransposons

**Figure 2:**
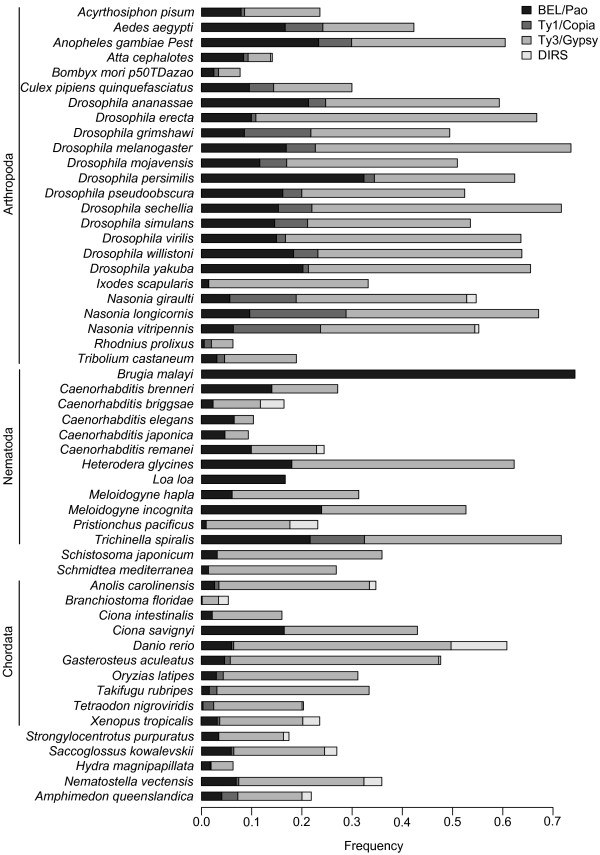
**Separation of LTR elements in different classes for each genome**. For each genome that contains BEL/Pao elements, we show the abundance of BEL/Pao, Ty1/Copia, Ty3/Gypsy and DIRS elements, based on our classification. The horizontal axis indicates the relative abundance of each element class in each genome, normalized to the interval (0,1). The difference in the length of each bar to the frequency of one reflects unclassified transposable elements. Their frequency is quite large in some genomes. The three most abundant phyla in our data set (arthropods, nematodes, chordates) are labeled.

To evaluate how important BEL/Pao elements are as a genome constituent, we compared their abundance to that of the other three LTR element subclasses. Figure 2 shows for each genome containing BEL/Pao elements the fraction of LTR elements that our de novo search identified, and that we were able to classify into one of the four LTR classes. BEL/Pao, Ty1/Copia, Ty3/Gypsy and DIRS elements are represented by different shades of grey in the figure. Ty1/Copia and Ty3/Gypsy are commonly considered the most abundant LTR elements, but our analysis invalidates that pattern. Although Ty3/Gypsy elements still are the most abundant class of LTR elements, they are followed by BEL/Pao elements, with Ty1/Copia a distant third. Specifically, while Ty3/Gypsy elements constitute an average of 68.9 percent of classifiable elements in a genome, BEL/Pao comprise 21.6 percent, and Ty1/Copia elements contribute an average of 6.7 percent. DIRS elements are a distant fourth with 2.8 percent, and they occur only in 19 genomes. In terms of absolute numbers, we identified 25,024 Ty3/Gypsy elements, 7861 BEL/Pao elements, 2445 Ty1/Copia elements and 1009 DIRS elements. The roundworm *Brugia malayi *is the only organism in which we only identified BEL/Pao elements (29 elements) and no elements belonging to the other three classes. Because our identification procedure of transposable elements purposedly excludes small element fragments, we cannot exclude that this organism may have contained elements from other families in the past. We also note that *B. malayi *is not the sole species containing only elements from one LTR subclass. For example, there are also 11 species which do not contain any BEL/Pao elements (Figure 2).

### Many different families

We next wished to group our identified BEL elements into families based on their sequence similarity on the nucleotide level. To this end, we pursued a graph-based approach. The nodes in the graph we studied are BEL/Pao elements. Edges represent sequence similarity between elements. The approach we pursued clusters the elements in the graph into families, using a fast and scalable unsupervised Markov cluster algorithm (MCL) for graphs, which is based on the simulation of stochastic flow on graphs [27,28] (see Methods for details).

**Figure 3:**
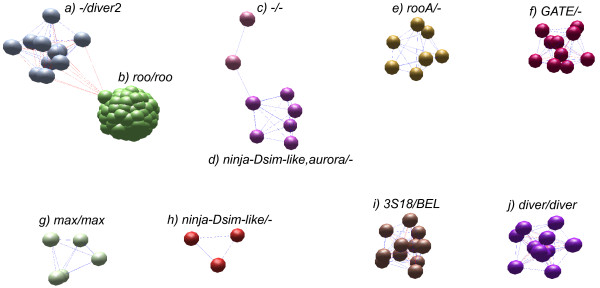
**Markov clustering of D. melanogaster BEL/Pao elements**. We clustered all 178 BEL/Pao elements from *D. melanogaster *into ten families based on their sequence similarity using the MCL algorithm [27] from within BioLayout [46]. We refer to these families as MCL families. A node in the graph represents one element. Edges represent nucleotide sequence similarity between two elements (see Methods for details). Elements clustered into the same family are shown in the same color. The absence of an edge between two elements, indicates that the elements do not share sufficiently high sequence similarity over at least 500 bp (see Methods). We compared the elements of the MCL families to previous annotation of these elements in the *D. melanogaster *genome and in Repbase Update. Each MCL family is labeled with two names separated by a slash. The left name is from the Drosophila genome annotation, the right name is from Repbase Update. Dashes '-' indicate that a family has not been previously annotated. Note that one family has two names: *3S18 *in the genome annotation and *BEL *in Repbase Update. Elements from family d) were previously annotated as belonging to two different families.

To validate the accuracy of our clustering approach, we first clustered only the BEL/Pao elements from the especially well-studied genome of *D. melanogaster*. We then compared our classification of the 178 *D. melanogaster *elements with (i) the annotation of elements in the genome sequence of *D. melanogaster*, and (ii) the *D. melanogaster *elements in Repbase Update [25]. Our approach resulted in the family classification shown in Figure 3, where the 178 BEL/Pao elements we identified form ten families labeled from a) to j). In this figure, each node represents an element and different node colors indicate different families. In the figure, we labeled elements in each family with the established names from the genome sequence (left name in each panel), and with the name of the most similar Repbase Update element (right name). Our classification is identical to the previous classification of these elements, with the exception of one family. This family (d)) contains two elements belonging to the previously described *ninja-Dsim-like *family, and one belonging to the *aurora *family. The other three elements in this family were not previously annotated. Additionally, we find three more *ninja-Dsim-like *elements that belong to the family in panel h) in our classification. In sum, the method we use classifies only one out of 178 (0.6 Percent) *D. melanogaster *BEL/Pao elements in a different family than previous annotations and divides one family. This low incidence of misclassification motivated us to apply the method to larger sets of elements.

We next applied our method to all 7,861 BEL/Pao elements we had identified. This resulted in 817 families with at least two elements and 908 singletons. We assigned a unique identifier to each family, which we report in additional file 4. Most (696) families have ten or fewer member elements. Only three families have more than 100 copies. One of these families is present in the fruit flies of the melanogaster subgroup (143 copies). This family corresponds to the *roo *family which has been described earlier to have a high copy number in *D. melanogaster *[29]. The family with the second highest copy number (207 copies) is present in the fruit fly *D. ananassae*, and that with the highest copy number (397 copies) in the carolina anole *Anolis carolinensis*, an arboreal lizard. These two families, however, do not share any similarity to families of known elements. Table 1 shows the ten families with the highest copy number.

**Table 1:** Top ten families with highest copy number

Element number	Taxa	Previously Assigned Name
397	*Anolis carolinensis*	
207	*Drosophila ananassae*	
143	*Drosophila melanogaster subgroup*	*roo*
94	*Drosophila melanogaster subgroup*	*diver2*
91	*Drosophila melanogaster subgroup*	
76	*Drosophila persimilis*	
69	*Drosophila willistoni*	
68	*Drosophila yakuba*	
68	*Drosophila ananassae*	
64	*Drosophila ananassae*	

Only 42 of the families (2.4 percent) we identified are not restricted to one genome, but contain elements from different genomes. Most (35) of these families occur in the *Drosophila melanogaster *and *obscura *groups. Four families have elements in two mosquito genomes and three families are distributed in the three *Nasonia *wasps. All these families are therefore restricted to closely related species. In addition to the family classification we just described, which clusters elements regardless of which genome they occur in, we carried out an analogous classification, but separately for elements within each genome. The two classifications do not differ dramatically. Additional file 5 describes the results of the species-specific classification.

### Phylogenetic relationship among BEL/Pao families

**Figure 4:**
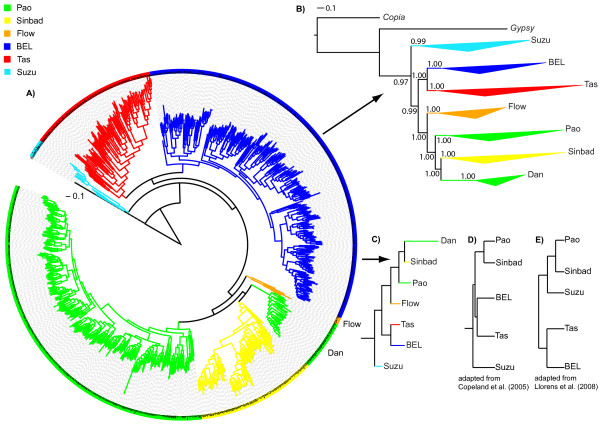
**Phylogenetic tree of BEL/Pao families**. The phylogenetic tree is based on the concatenated amino acid sequence of the three protein domains protease, reverse transcriptase, and integrase. The tree is based on 893 transposable element families for which we could construct a consensus sequence for all three domains. In addition to data from these families, we used in this analysis all BEL/Pao elements from Repbase Update [25] where we could identify all three domains (total of 92 elements). Some of these Repbase Update elements are from species whose genomes we did not analyze here. Furthermore, we included 16 elements from the Gypsy element Database GyDB [23], in order to associate clades in the tree to previously described subclades of the BEL/Pao element class. A list of these elements is found in additional file 2. Different colors indicate major clades. A) Same phylogenetic tree as in A) but with major clades collapsed into triangles indicating superfamily divergence and statistical support values for the branches. We constructed the tree using PhyML with an approximate likelihood ratio test to estimate the statistical support of the tree topology [31]. This statistical support is indicated by the numbers at the branches which range from 0 (least significant) to 1 (highly significant). All branches leading to the major clades have very high support. C) Same phylogenetic tree as in A) but without divergence triangles, and branches are reorganized to facilitate comparison with trees in D) and E). D) and E) Phylogenetic relationship of BEL/Pao clades as described by Copeland et al. [16] and Llorens et al [23], respectively. High resolution tree and graphic files are available from the authors upon request.

We next wanted to use our large data set to validate the existing classification of BEL/Pao elements into the previously identified superfamilies BEL, Tas, Suzu, Sinbad and Pao. To this end, we used the protease, reverse transcriptase and integrase domains of each element. We constructed a consensus sequence for each domain in each of our element families or used the domain sequences themselves for elements that were singletons (see methods for details). We then used these (consensus) sequences to construct a multiple alignment of each of the domains using Mafft [30] (three alignments in total). Subsequently, we concatenated these alignments and constructed a phylogenetic tree from them using PhyML_aLRT [31], a version of PhyML [32] that incorporates an approximate likelihood ratio test to estimate the statistical support of the tree topology. In this tree reconstruction, we included as outgroups the domains of the canonical sequence from the *Gypsy *and *Copia *elements from *D. melanogaster*, as given by Repbase Update [25]. The consensus sequences for each of the families and domains we identified are listed in additional file 6. Additionally, we provide the relevant alignments in additional file 7. We defined superfamilies in this tree as major, deep-branching clades, which are easily identifiable based on the tree structure, and further discussed below. The complete phylogenetic tree is shown in Figure 4A, and in additional file 8 with partly collapsed clades, and representative species names assigned to branches. Figure 4B shows an additional version of the tree with collapsed major clades. In Figure 4B the triangles represent the divergence between the elements within each major clade, with long triangles indicating great divergence. Numbers next to tree branches indicate the statistical support for a clade ranging from 0 (least significant) to 1 (highly significant). Figure 4C shows the same tree for the purpose of comparing it to previously proposed phylogenetic trees of BEL/Pao elements. For clarity, this panel does not show the divergence within each major clade, and the order of the branches is reorganized to ease the comparison to these previous phylogenetic trees (shown in Figure 4D and 4E) [16,23]. To avoid confusion, we note that if we refer to BEL/Pao elements below, we mean the entire subclass of LTR elements we studied. If we refer to only BEL or Pao elements, we mean the BEL and Pao superfamilies, respectively.

Previous studies identified five different superfamilies of BEL/Pao elements which are named after the first identified element in the subclade: BEL [33], Tas [18], Suzu [14], Sinbad [16] and Pao [11]. Two previous phylogenetic studies both found that BEL and Tas elements formed one clade and Sinbad and Pao another one. In both studies, Suzu elements alone formed a third clade [16,23]. However, the trees that emerged from these two studies differ in one major way. Copeland et al. identified elements belonging to the Suzu superfamily as more distantly related to the other four clades than these clades are to each other (Figure 4D) [16]. In contrast, the study by Llorens et al. grouped the Suzu superfamily with the Pao/Sinbad clade (see Figure 4E) [23].

### Two new superfamilies of BEL/Pao elements

Our phylogenetic analysis shows some similarities to the previous studies. For example, we find that Pao and Sinbad form part of the same clade as do BEL and Tas. Similar to what Copeland et al. (2005) [16] reported, the elements from the Suzu family are more distantly related to the other clades than these clades to each other. However, there are two major differences. First, we found that the Pao superfamily groups into two separate clades (green clades in Figure 4A and 4B). We kept the name Pao for the larger of these clades, because it contains the original *Pao *element identified in *Bombyx mori *[11]. We propose the new name Dan for the second, smaller clade, because we have identified it in the zebrafish *Danio rerio*, and it currently only contains elements from this organism. Second, we identified a new superfamily (discussed below), for which we propose the name Flow. Table 2 shows the number of families and elements per superfamily and additional file 9 the minimal, maximal, and median element and LTR sequence lengths for each superfamily. We note that we could have classified our data into many more superfamilies, but our aim was not to proliferate the number of superfamilies unnecessarily, while preserving previous classifications as much as possible. Fortunately, the clear partitioning of our family tree into few major clades made this task easy and unambiguous. We also note that our phylogenetic analysis is based on more than 40 times more sequence data than previous analyses.

**Table 2:** Number of elements, families, species and phyla for every superfamily

Superfamily	Elements	Families	Species	Phyla
Pao	1723	323	14	1
Sinbad	791	85	9	5
Dan	85	33	1	1
Flow	83	5	3	2
Tas	270	104	17	5
BEL	2955	333	21	2
Suzu	30	10	5	3

Table 3 shows the average amino acid similarity in percent between the consensus sequences within a superfamily and among different superfamilies. Not unexpectedly, the average similarity of elements within one superfamily (diagonal in Table 3) is generally higher than the average similarity of elements in different superfamilies (off-diagonal in Table 3). The only exception are elements belonging to the Tas superfamily. They are on average slightly less similar to each other than elements in some different families, e.g., elements in the Dan and Sinbad superfamily. The average similarity within one superfamily varies between 37.4 percent for the sequences belonging to the Tas superfamily, to 63.53 percent between sequences belonging to the Dan superfamily. The average similarity between different superfamilies ranges from 26.51 percent between elements from the Tas superfamily and the Suzu superfamily, to 39.07 percent between elements from the Dan and Sinbad superfamilies.

**Table 3:** Average amino acid similarity between BEL/Pao superfamilies

	BEL	Dan	Flow	Pao	Sinbad	Suzu	Tas
**BEL**	46.73	35.44	35.52	34.65	34.25	31.06	32.04
**Dan**		63.53	35.29	37.80	39.07	31.06	31.62
**Flow**			45.48	37.42	35.37	29.96	31.09
**Pao**				51.84	37.84	30.89	31.26
**Sinbad**					49.24	30.42	30.78
**Suzu**						52.54	26.51
**Tas**							37.40

### Distribution of superfamilies

The number of species and phyla covered by each superfamily is shown in Table 2 and a list of phyla covered by each superfamily can be seen in additional file 10. Some superfamilies are restricted to one phylum. The Dan and Pao superfamilies have the most restricted host range and occur only in the fish *Danio rerio *and in arthropods, respectively. Pao elements occur mainly in the different *Drosophila *and mosquito genomes, with the exception of a few elements that are present in the parasitoid wasp *Nasonia vitripennis*.

Other superfamilies are present in different phyla and in a wider range of host organisms. An example is the Tas superfamily, which is present in Cnidaria, Nematoda, Arthropoda, Hemichordata, and Porifera. It also occurs in different host organisms within all phyla, e.g., in almost all our nematode genomes (11 genomes). Some superfamilies are present in a wide range of phyla but all species are restricted to a certain habitat type, such as aquatic habitats for the Suzu superfamily.

We note that our study is the first to identify elements of the BEL/Pao subclass in the subkingdom parazoa. These elements fall into the BEL and Tas superfamilies.

### Elements of the Flow superfamily

The five families belonging to the new superfamily we discovered are all present in lower animals that live in aquatic habitats, hence the name Flow. One of Flow's families occurs in the starlet sea anemone *Nematostella vectensis *(7 copies), and two families occur in the fresh water hydra *Hydra magnipapillata *(55 copies). Both species belong to the phylum Cnidaria. We found the other two families in the planarian *Schmidtea mediterranea *(21 copies), phylum Platyhelminthes. The elements are between 3303 to 16272 base pairs long with an average of 7452 base pairs.

The five families of the Flow superfamily are not highly similar to one another. Specifically, the amino acid similarity ranges from 33.89 percent between the consensus sequence of *N. vectensis *and one of the consensus sequences from *S. mediterranea*, to 53.95 percent between the two consensus sequences from *S. mediterranea*. The average similarity is with 45.48 the second lowest similarity within a superfamily. The average similarity to the other BEL/Pao superfamilies varies from 29.96 percent (Suzu superfamily) to 37.42 percent (Pao superfamily).

### Highly similar elements in closely related species

We used blastn[34] to compare each BEL/Pao element against all other 7,860 elements to search for possible signs of horizontal transfer of BEL/Pao elements between our studied species. If an element is only transferred vertically from parent to offspring, we expect to find no high similarity between elements from distantly related species. In contrast, highly similar elements occurring in very distantly related species might indicate horizontal transfer of an element between these species. In this analysis, we required an identity of at least 80 percent over at least 20 percent of the length of an element, but at least over 300 bp. This criterion is a liberal threshold for recent transfer events and allows for some divergence of sequence since the transfer. Using this criterion, we found no signs of similar sequences in very different species. The only similar sequences in different species occurred in closely related species within the same genus, i.e., in fruit flies of the genus *Drosophila*, in parasitoid wasps of the genus *Nasonia*, and in roundworms of the genus *Meloidogyne*.

Previous work had identified a horizontal transfer of a BEL element between the fly *Drosophila ananassae *and the endosymbiont Wolbachia [35]. Motivated by this observation, we attempted to identify further possible horizontal transfers to endosymbiont genomes and viruses through a blast search [34] of our consensus sequences for the concatenated protease, reverse transcriptase, and integrase domains for each of the seven superfamilies against the non-redundant protein and nucleotide databases of GenBank. The BEL element in the endosymbiont Wolbachia was the only one we found, suggesting that such horizontal transfer is not widespread for BEL/Pao elements.

## Discussion

### Many BEL/Pao elements in metazoan genomes

We identified 7,861 BEL/Pao elements in 53 out of our set of 62 non-mammalian metazoan genomes. This is the first time genome-wide abundances of BEL/Pao elements are reported. Previous studies identified around 160 families of BEL/Pao elements but in most cases the copy number of a family was not reported. The genomes we studied span a wider range of species than previously analyzed for BEL/Pao elements, and so does the distribution of BEL/Pao elements we identified. For example, we were able to identify 24 BEL/Pao elements in the sponge *Amphimedon queenslandica*, a species belonging to the metazoan subkingdom parazoa. All previous BEL/Pao elements had only been identified in the subkingdom eumetazoa. Previous studies reported the lack of BEL/Pao elements in mammalian genomes [16]. We screened eleven mammalian genomes for BEL/Pao elements but did not identify any elements. This observation supports the view that BEL/Pao elements are not present in mammalian genomes. This might be explained by stochastic loss of BEL/Pao elements in early mammalian evolution. Alternatively, the BEL/Pao subclass might only have originated after the origin of the mammalian clade. However, the presence of BEL/Pao elements in species belonging to both metazoan subkingdoms makes this unlikely. Most previous studies identified BEL/Pao elements in the three phyla Nematoda, Arthropoda and Chordata. Most of our elements also come from these three phyla because 53 of our 62 sequenced genomes belong to these phyla. We identified BEL/Pao elements in 46 of these genomes (86 percent), including 18 genomes where, to our knowledge, no BEL/Pao elements had been identified so far. This shows that BEL/Pao elements are more abundant than previously thought. We found BEL/Pao elements in seven of the remaining nine genomes, which do not belong to either of the three phyla Nematoda, Arthropoda and Chordata. In two of these seven species no BEL/Pao elements had been identified so far. The wide distribution of BEL/Pao in different metazoan phyla and the occurrence in both metazoan subkingdoms makes it likely that BEL/Pao elements evolved during early metazoan evolution.

The number of BEL/Pao elements we identified in different species varies from zero to more than 2,500 copies in *A. aegypti*. The high number in *A. aegypti *is not surprising because the genome of this species is with 1.3 Gb our largest studied non-mammalian genome, and it has been previously reported that more than 50 percent of the *A. aegypti *genome consists of transposable elements [26]. More generally, transposable elements in *A. aegypti *are a major factor for its genome size increase compared to related mosquito and fruit fly species [26]. The reason why we find such a big difference in the abundance of BEL/Pao elements in some species remains unknown (as it is for many other transposable elements).

Several factors may influence variation in TE numbers. They include stochastic loss of transposable elements [1], transposition bursts in response to environmental stressors [36], and differences in effective population sizes that render selection against the deleterious effects of transposable elements more or less effective [10]. Additionally, we note that our transposable element identification approach might only give a lower bound for the number of BEL/Pao elements in a genome. The reason is that genome sequences differ in their quality. Many genomes are represented by many small sequence contigs instead of one sequence per chromosome. This might prevent the identification of some elements at the end of a sequence contig. However, we note that this cannot be the only cause for the differences we find. For example, the genome sequences of *A. aegypti *and *D. melanogaster *are available as contigs with a median length of 683 kb and 10,805 kb, respectively. Even though the genome of *D. melanogaster *has longer contigs than *A. aegypti*, it contains fewer BEL/Pao elements (178 versus 2524 elements, respectively).

Overall, BEL/Pao elements are the second most abundant class of LTR retrotransposons in the genomes we studied, a pattern that also holds for most of our study genomes individually. Only elements belonging to the Ty3/Gypsy class are, on average, more abundant. By comparison, Ty1/Copia and DIRS elements account for only a small fraction of LTR retrotransposons (6.7 and 2.8 percent, respectively). Only some genomes, such as that of *Brugia malayi *and the two *Nasonia *species, show a different distribution of LTR element classes. *B. malayi*, a causative agent of the tropical disease lymphatic filariasis, contains only BEL/Pao elements and no elements from the other classes. The *Nasonia *species on the other hand, harbor all four classes and BEL/Pao elements are only the third most abundant class (11.7 percent over all three species). Our observations are broadly consistent with previous studies. For example, one study in *A. gambiae *[24] and one in *B. mori *[17] report the Ty3/Gypsy class to be the most abundant LTR class in these two organisms, followed by BEL/Pao and Ty1/Copia. Another study focused on LTR elements in *C. elegans *[37], and it found the BEL/Pao class to be the most abundant, followed by the Ty3/Gypsy class. It did not identify any Ty1/Copia elements [37]. Our results agree with this finding.

While previous studies concentrated on elements from one species, we analyzed elements from multiple species. However, our data set still only represents a small fraction of all metazoan species. The NCBI's genome sequencing project site lists 88 non-mammalian metazoan genomes as "in progress" http://www.ncbi.nlm.nih.gov/genomes/leuks.cgi. Some of these species cover phyla which have not been studied so far with respect to BEL/Pao elements. Therefore, the results of our study will likely be extended with every new genome release.

### A ten-fold increase in family number

We clustered our BEL/Pao elements into 1,725 families (817 multi-element families and 908 singletons) which is more than a ten-fold increase in family number compared to the approximately 160 BEL/Pao families known so far. Part of this increase can be explained by the 251 families identified in the 20 previously not analyzed genomes. However, the bulk of this increase in family number comes from newly identified families in previously studied species. For example, we identified two previously unknown families with a copy number of more than 200 elements.

One potential explanation for the high family number might be that our clustering approach divided the elements from one species into too many small families. However, our analysis of the well-studied and well-annotated fruit fly *D. melanogaster *genome argues against this possibility. This analysis finds that the ten families we identify are largely congruent with previous annotation. The only exception are elements belonging to the *ninja-Dsim-like *family (nine elements). Our clustering approach divided this family into two families. Additionally in one of the two *ninja-Dsim-like *families, we find an element from the *aurora *family. However, this change in annotation affects only one in 178 elements (0.6 percent). The *aurora *element is the only element of that family in *D. melanogaster*, raising the possibility that one of the *ninja-Dsim-like *elements was falsely annotated as an *aurora *element. Indeed, the alignment of all annotated *ninja-Dsim-like *elements in *D. melanogaster *shows a high average sequence divergence (in both *ninja-Dsim-like *families the elements have an average similarity of 64 percent) and the *aurora *element is very similar to large parts of some of the *ninja-Dsim-like *elements.

Overall, the comparison of our families to the previously annotated TEs in *D. melanogaster *shows that our clustering does not separate the elements into too many small families. Our approach correctly classified 177 out of 178 elements. We therefore believe that the high number of BEL/Pao families we identified is a faithful reflection of actual BEL/Pao element diversity.

### Major subclades separated early

Previous studies, which were based on fewer than 25 BEL/Pao elements, identified five superfamilies of BEL/Pao elements: Pao, Sinbad, BEL, Tas and Suzu [16,23], named after the first element identified in each superfamily. Our phylogenetic tree is based on 893 BEL/Pao families and it also identified these five known superfamilies. Additionally we found one completely new superfamily (Flow), and a deep separation of the Pao superfamily into the two highly divergent superfamilies Pao and Dan.

We separated the Dan superfamily from the Sinbad superfamily for two reasons. First, one element from the Dan clade was previously annotated to belong to the Pao superfamily. In contrast, we find that the Dan clade is more closely related to the Sinbad superfamily. The best way to resolve this conflict, in our view, is to propose Dan as a second superfamily. Second, because the Sinbad superfamily also contains elements from *Danio rerio*, the Dan clade is not simply a subbranch of the Sinbad superfamily that contains all elements from *Danio rerio*. In sum, we think that the Dan clade should be viewed as a separate superfamily. It is very likely that new elements of this superfamily will be identied in genomes that we did not study.

The superfamily tree topology we find differs somewhat from previously reported topologies [16,23]. We find a very high statistical support for our tree topology (see Figure 4B). This high support makes it unlikely that our tree topology is not correct, especially as it is also supported by many more sequences than any of the previous studies. Additionally the average sequence similarity of elements within one superfamily almost always shows greater amino acid sequence similarity than elements in different superfamilies, which supports our classification into superfamilies.

Most (656 of 893) of our families belong to the Pao or BEL superfamily. This is not surprising because these superfamilies are mainly restricted to arthropod genomes and 27 of our genome sequences (44 percent) belong to this phylum. The other five superfamilies show a more diverse distribution of host genomes. Here, the host genomes come from more than one phylum and, for the Tas and BEL superfamily, even from both metazoan subkingdoms.

The variation in the host species range that we observe has several candidate explanations that our data cannot resolve. On the one hand, BEL/Pao superfamilies might have been lost in some phyla. On the other hand, horizontal transfer of BEL/Pao elements between species belonging to different phyla might be at work. For example, if BEL/Pao elements originated in the eumetazoa, a horizontal transfer event to *Amphimedon queenslandica *might explain the presence of BEL/Pao elements in the parazoa. We did not find any evidence of recent horizontal transfer between distantly related species, because no such species contain highly similar BEL/Pao elements. This, however, does not exclude the possibility of ancient horizontal transfer events. To identify such transfer events is beyond the scope of our study. But regardless of whether element loss or horizontal transfer explains the current BEL/Pao element distribution, BEL/Pao elements probably originated early in metazoan evolution. This is because BEL/Pao elements occur in both metazoan subkingdoms, and in a wide range of host species from different phyla.

### Flow, a new superfamily of BEL/Pao elements

We found a new BEL/Pao superfamily (Flow) which consists of five families and a total of 83 elements present in lower animals. We identified three of these families in two species belonging to the phylum Cnidaria (*Hydra magnipapillata*, *Nematostella vectensis*), and the other two families in a species belonging to the phylum Platyhelminthes (*Schmidtea mediterranea*). In both phyla, Cnidaria and Platyhelminthes, only eleven and two BEL/Pao elements, respectively, had been identified before [16,23,38]. The five Flow families are quite diverse (average similarity 45.48 percent). This is not surprising, because they occur in very distantly related species. The new superfamily supports and strengthens the view that BEL/Pao elements arose in early metazoan evolution, because none of its families are present in one of the three well studied phyla Nematoda, Arthropoda, and Chordata.

## Conclusions

We identified 7,861 BEL/Pao elements in 53 metazoan genomes, making the BEL/Pao elements the second most abundant class of LTR retrotransposons in metazoan genomes. The elements we identified can be divided into 1,725 families based on the similarity of their nucleotide sequence. Our analysis increases the number of known BEL/Pao families by more than ten-fold. These families can be separated into seven superfamilies based on their phylogenetic relationship. Five of these superfamilies have been known previously, one new superfamily emerges from a highly divergent existing superfamily, and one superfamily is completely new. BEL/Pao elements are present in both metazoan subkingdoms, which suggests that they arose during early metazoan evolution.

## Methods

### Element identification

We downloaded a total of 73 non-mammalian metazoan genomes from the NCBI's eukaryotic genome sequencing project site http://www.ncbi.nlm.nih.gov/genomes/leuks.cgi with a sequencing status of "Assembly" or "Complete" as of August 25, 2010. If more than one genome sequence was available for one species, we used the sequence that covered most of the genome. Functional LTR elements are at least 5 kb long. To reduce the search time and avoid falsely identifying elements, we excluded contigs from the genome sequences with a length of less than 10 kb from our analysis. The available genome sequence for eleven genomes comprised fewer than 1.5 Mbps. We also excluded these genomes from our analysis because they are too short for a genome-scale analysis. A complete list of all genomes we analyzed is present in additional file 2.

Although previous studies did not identify any BEL/Pao elements in mammalian genomes, we downloaded eleven mammalian genome sequences from the NCBI's eukaryotic genome sequencing project site to validate this observation: *Bos taurus*, *Canis lupus familiaris, Equus caballus, Monodelphis domestica, Mus musculus, Ornithorhynchus anatinus, Rattus norvegicus, Ailuropoda melanoleuca, Cavia porcellus, Lama pacos, Pteropus vampyrus*.

We used a combination of two de novo identification algorithms to identify all LTR elements in our set of genomes. Specifically, we applied the two de novo LTR identification algorithms LTRharvest [39] and find_ltr [40] to identify all possible LTR elements in our genomes. Subsequently, we separated our de novo identified elements into the four LTR classes Ty1/Copia, Ty3/Gypsy, BEL/Pao and DIRS, merged the two sets of BEL/Pao elements from each algorithm into one set, and assigned an identifier to each element. We now explain important details of this procedure.

LTRharvest and find_ltr both search for structural features, such as LTR sequences, to identify full length LTR elements in a genome sequence. Among all de novo LTR identification programs LTRharvest and find_ltr give the best results with regard to the number of true LTR elements detected [41]. However, the number of false positives can also be high for both programs. Therefore we accepted only candidate elements for further analysis that contained at least one functional domain known to be present in LTR retrotransposons and at least one open reading frame longer than 300 bp. To identify these domains we used hidden Markov models [42] obtained from Pfam [43] (Asp_protease, Peptidase_A17, RVT_1, RVT_2, rve, Integrase_Zn, GP36, Retrotrans gag, Integrase, Integrase_Zn, TLV_coat) and used Hmmer http://hmmer.janelia.org to compare these Pfam domains to our de novo candidate elements. Only if Hmmer had found at least one domain with an E-value smaller than 0.01 in an element, we kept the element for further analysis.

We used LTRharvest [39] with the minimum distance between two LTR sequences (option-mindistltr) set to 2000 base pairs and allowed overlapping hits (option-overlaps). We used find_ltr with default values. Both programs, but especially LTRharvest, return overlapping and nested elements. Where several elements were nested, we only took the innermost element, because it probably represents the younger element. Where elements overlapped, we randomly chose one of the elements for further analysis.

To divide our elements from both de novo sets into Ty1/Copia, Ty3/Gypsy, BEL/Pao and DIRS classes, we constructed specific hidden Markov models for each class. To this end, we downloaded all canonical LTR sequences, prototypic sequences that either represent consensus sequences or a sequence example for a TE family, for each of the four classes from Repbase Update, a database containing repetitive DNA elements in eukaryotes (478, 941, 106 and 68 sequences for Ty1/Copia, Ty3/Gypsy, BEL/Pao and DIRS, respectively). We then used the Pfam hidden Markov models [43] for the domains listed in the previous paragraph, and identified these domains in all sequences from a given class using Hmmer (http://hmmer.janelia.org; E-value<0.0001). For each LTR class we took all identified domain sequences and aligned the sequences belonging to the same domain using Mafft [30], checked the alignments manually for obvious errors, and constructed a new hidden Markov model using Hmmer. We next used Hmmer to compare each of the new candidate elements that we had identified against these class-specific hidden Markov models [42]. For each candidate element and each element class we obtained in this way an E-value that reflects how well the element matches the class. We assigned the candidate element to an element class if it matched this class with the smallest (most significant) E-value among all four classes we matched it with. If we did not find a model with an E-value below 10^-20^, we did not classify the element. By the time we had finished this (time-consuming) analysis, a larger set of specific hidden Markov models for each of the four element classes than our set became freely available [22]. We tested if this new set improved the classification substantially, which was not the case (results not shown).

At this stage, our analysis had created two sets of elements classified as BEL/Pao elements, one set from each of our de novo searches. For each species, we then merged these two BEL/Pao sets into one set of elements according to the following rules: If only one of the search algorithms had identified an element at a given genome position, this element was used for the final set. If both algorithms had identified an element at the same position, we took the element identified by find_ltr, because in our experience find_ltr identified the LTR start and end positions more accurately. If both algorithm had identified an element within 20 bp of each other, we took the element identified by find_ltr. Otherwise, if both algorithms had identified an element, and if these elements were overlapping but their start and end positions differed by more than 20 bp, we took the element which had a length between 2,000 to 15,000 bp. We chose these length thresholds, because based on known elements they are lower and upper bounds for full length elements. If both elements were within this length range, we randomly chose one element. If neither element fulfilled this length criterion, we eliminated the element from further analysis. This occurred only for 0.7 percent (53 out of 7914) of elements we analyzed.

In sum, our final set of BEL/Pao elements for each species is a merged set from de novo identified elements by LTRharvest [39] and find_ltr[40].

### Family identification

The next stage of our analysis began with the BEL/Pao elements we had identified, and grouped them into different families. To this end, we used the Markov cluster (MCL) algorithm, a fast and scalable unsupervised Markov clustering algorithm for graphs based on simulation of stochastic flow in graphs [27,28]. This algorithm subdivides a graph whose nodes are transposable elements, and whose weighted edges reflect sequence similarity among elements, into subgraphs. The algorithm defines a family based on the higher connectivity between elements of one family than to elements of a different family. It can therefore overcome the limitations of a fixed similarity threshold (usually 80 percent) for classifying sequences into families. Such a threshold can lead to an inconsistent classification of families, because some element pairs within a family may be more similar than the threshold, whereas others may be less similar. Also, the mere choice of a single threshold lends an element of arbitrariness to the classification procedure, which our approach avoids. Similar clustering approaches were also used to identify protein families between different species (for example [28,44]), and for the reconstruction of a cyanobacterial tree from conserved protein families [45]. To create a graph out of our BEL/Pao elements, we used a procedure suggested by the author of MCL [27]http://www.micans.org/mcl/man/clmprotocols.html. To be able to compare our clustering to previously annotated families for selected species, we additionally carried out clustering for each species separately. We next describe our procedure in greater detail.

We first carried out an all-against-all nucleotide sequence comparison of all elements using Blast[34], and recorded all matches with an overlap of at least 500 bp (which corresponds to an average E-value cutoff below *e*^-5^). If any pair of sequences matched over more than one stretch of nucleotides, and thus showed two or more matches, we only used the match with the lowest E-value. For each pair of elements with a match, we then converted the E-value into a similarity score in the interval 0[200] by calculating the negative decadic logarithm of the E-value, and assigning all logarithmically transformed scores greater than 200 a value of 200, thus effectively truncating the score distribution at highly significant E-values. We then normalized these scores to the interval 0[1]. Thus, a match with an E-value <*e*^-200 ^received the score one, and a match with an E-value of 1 received the score zero.

In the graph-based grouping procedure we used, our elements correspond to nodes and the similarity score between two elements correspond to the edge weight between the two nodes. We loaded the scores into BioLayout [46], a 3D graph visualization tool, and started MCL [27] from within this tool. The inflation option of the MCL algorithm affects the cluster granularity. We tested different inflation values on our set of 178 BEL/Pao elements from the well studied *Drosophila melanogaster *genome, and compared the resulting clustering to the genome annotation. We found that an inflation value of 4.0 and a pre-inflation value of 3.0 best reproduced the known *D. melanogaster *families. We also used these values for our clustering analysis among genomes. Additionally, we set the smallest detectable cluster size to one. BioLayout assigns an arbitrary color to each element family, and paints the nodes belonging to a family in that color.

Changing the length threshold for our blast matches above would influence the number of element pairs we find, and therefore the number of edges in our graph. A lower length threshold would increase the edge number, whereas a higher threshold would decrease it. We explored different length thresholds and did not find qualitative differences between the clustering of the elements. Also, the number of families with multiple elements did not vary much. By decreasing the length threshold progressively, however, our approach identified fewer singletons (families with only one member), because increasing numbers of singletons got added to existing multi-element families. Conversely, increasing the length threshold, results in more singletons, as families become more and more fragmented. These new singletons typically are the most diverged elements in a family.

### Sequence alignment and phylogenetic reconstruction

We next describe how we constructed the phylogenetic tree that helped us classify elements into superfamilies. Our procedure had three steps. In the first, we defined, separately for each family, a consensus sequence for each of the protease, reverse transcriptase and integrase domains (three consensus sequences per family). Second, we used these consensus sequences to produce a multiple sequence alignment of elements in all families. Third, we constructed a phylogenetic tree from this alignment. We now describe important details of each step.

In the first step, we constructed for each of the families we had identified multiple alignments of the protease, reverse transcriptase and integrase domains using Mafft version 5 [30]. To this end, we used the amino acid sequences of the domains of all elements within one family. From the resulting alignments, we constructed a consensus sequence for each domain using the most common amino acid at any given position. If two or more amino acids were equally most common, we chose one of them randomly.

Additionally, we required that an amino acid had to be present (i) in at least one third of the sequences in the alignment, and (ii) in at least two sequences. If we could not identify a consensus amino acid at any one position based on these rules, we used the letter 'X' in the consensus sequence at that position. If the average pairwise similarity in the multiple alignment of one domain was below 70 percent and/or more than 5 positions in the consensus sequence correspond to an 'X', we validated the alignment and consensus sequence manually.

In the second step, we used all families where a consensus sequence for all three domains was available (893 families in total, or 51.8 percent of all our families). To be able to also include previously identified elements and elements from species we had not analyzed, we included all BEL/Pao elements from Repbase Update [25] and from the Gypsy Database (GyDB) [23] where we were able to identify all three domains (92 and 16 elements in total, respectively). Using these elements and the families we had identified we constructed a multiple alignment for each domain (three alignments in total) based on the consensus sequences for each family described in the preceding paragraph. We then concatenated these three alignments into one alignment in the order in which these domains occur in BEL/Pao elements (protease, reverse transcriptase and integrase) [16]. We concatenated sequences only after the alignment instead of before aligning them to avoid falsely aligning sequences from different domains.

Based on the concatenated multiple alignment, we then computed a phylogenetic tree of BEL/Pao elements using PhyML_aLRT [31] a version of PhyML [32] that incorporates an approximate likelihood ratio test to estimate the statistical support of the tree topology. This approach is superior to a bootstrap calculation with respect to accuracy and power, and it is computationally much more efficient [31]. The method assigns to each branch a statistical significance ranging from 0 (least significant) to 1 (highly significant). We used the default options of PhyML_aLRT with the JTT matrix for amino acid substitutions, the proportion of invariable sites set to zero, and with only one category of substitution rate [32]. We chose the χ^2^-based parametric branch support for approximate likelihood ratio tests [31]. As outgroups we used the domain sequences from the *Copia *and *Gypsy *element in *Drosophila melanogaster*, as given by Repbase Update [47,48].

We then separated our BEL/Pao families into superfamilies based on the major clades in the phylogenetic tree. Using the protdist program from the PHYLIP package [49] we calculated the average percent similarity of sequence pairs within a superfamily, as well as for sequence pairs whose members belonged to different superfamilies.

## Authors' contributions

NC carried out the research. NC and AW designed the study and wrote the manuscript. All authors read and approved the final manuscript.

## Supplementary Material

Additional file 1 **Nucleotide sequence of all identified BEL/Pao elements**. Nucleotide sequence of all BEL/Pao elements that we identified de novo in fasta format. Each record (element) has a unique fasta identifier consisting of three parts: (i) element identifier, (ii) internal family identifier as listed in additional file 4, and (iii) species identifier as listed in additional file 2. All identifier are joined by the underscore symbol '_'. For example, the identifier 5_NC-3_1 represents element 5 belonging to family NC-3 and is present in species 1 (*Drosophila melanogaster*). Click here for file

Additional file 2 **List of used genomes, Repbase Update, and Gypsy Database elements**. All used metazoan genomes are listed together with an internal identifier. Additionally we give the current URL from which the genome sequence can be accessed, the accession numbers, number of sequences of the genome included in our analysis, overall number of nucleotides, the number of BEL/Pao elements we identified in that genome, the number of different BEL/Pao families we identified in that genome, and to which subkingdom/superphylum/phylum the species belong. Additionally we list all mammalian genomes we used and all genomes we excluded from our analysis. Furthermore, we give the name of all BEL/Pao elements from Repbase Update and from the Gypsy Database, together with the species name they occur in, and an internal identifier. The internal identifiers are also used for the nucleotide sequences in additional file 1 and in the sequence alignment of additional file 7. Click here for file

Additional file 3 **BEL/Pao copy number per Mbps**. A) The histogram shows the number of genomes containing a given copy number of BEL/Pao elements per Mbps. The inset shows the number of genomes containing between zero and one BEL/Pao elements per Mbps. The eight genomes containing more than one BEL/Pao element per Mbps come from either fruit fly or mosquito species. B) Relationship between total copy number and copy number per Mb for each genome. Each point in the graph represents one genome and shows the total BEL/Pao copy number and the copy number per Mbps. Note the logarithmic scale on both axes. Click here for file

Additional file 4 **BEL/Pao families and their copy numbers**. For each BEL/Pao family we list the copy number, species, and the superfamily in which they are present. Families that we did not use in the phylogenetic tree construction are not assigned to a superfamily. Click here for file

Additional file 5 **Species specific family classification**. We describe the differences and agreements between the among-species family classification as used in the main text and the within-species family classification. Click here for file

Additional file 6 **Amino acid sequences of consensus domains**. A fasta file with all amino acid sequences for the domain consensus files we used for the phylogenetic tree reconstruction. The identifier consists of the family identifier (see additional file 4) and the domain name, for example NC-1_integrase represents the consensus sequence of the integrase domain of family NC-1. Click here for file

Additional file 7 **Multiple alignment of family domains**. The multiple alignment of 893 concatenated domain sequences. The phylogenetic tree is based on this multiple alignment. Click here for file

Additional file 8 **Phylogenetic tree of BEL/Pao elements with species names**. The Figure shows the same tree as in Figure 4 in the main text but with the species names shown in which the elements occur. If a clade of elements contained only element families from the same species or from very closely related species (e. g. mosquito species), the clade was collapsed to reduce the size of the tree. All species names are shown at the leaves of the tree. If all species in one clade of the tree belonged to the same genus, such as the genus Drosophila, only the genus name is shown, with the number of species in brackets. Major clades are highlighted in different colors. Click here for file

Additional file 9 **Structural information of superfamilies**. The table shows the minimal, maximal, and median element lengths (in basepairs) and the minimal, maximal and median LTR length for the major superfamilies we identified. Click here for file

Additional file 10 **List of phyla covered by each superfamily**. The table shows the phyla in which BEL/Pao superfamily members were identified. Click here for file
