# Supplementary figures and images for: BEL/Pao retrotransposons in metazoan genomes
Source: BMC Evol Biol. 2011 Jun 4;11:154. doi: 10.1186/1471-2148-11-154 (PMC3118150; doi:10.1186/1471-2148-11-154)

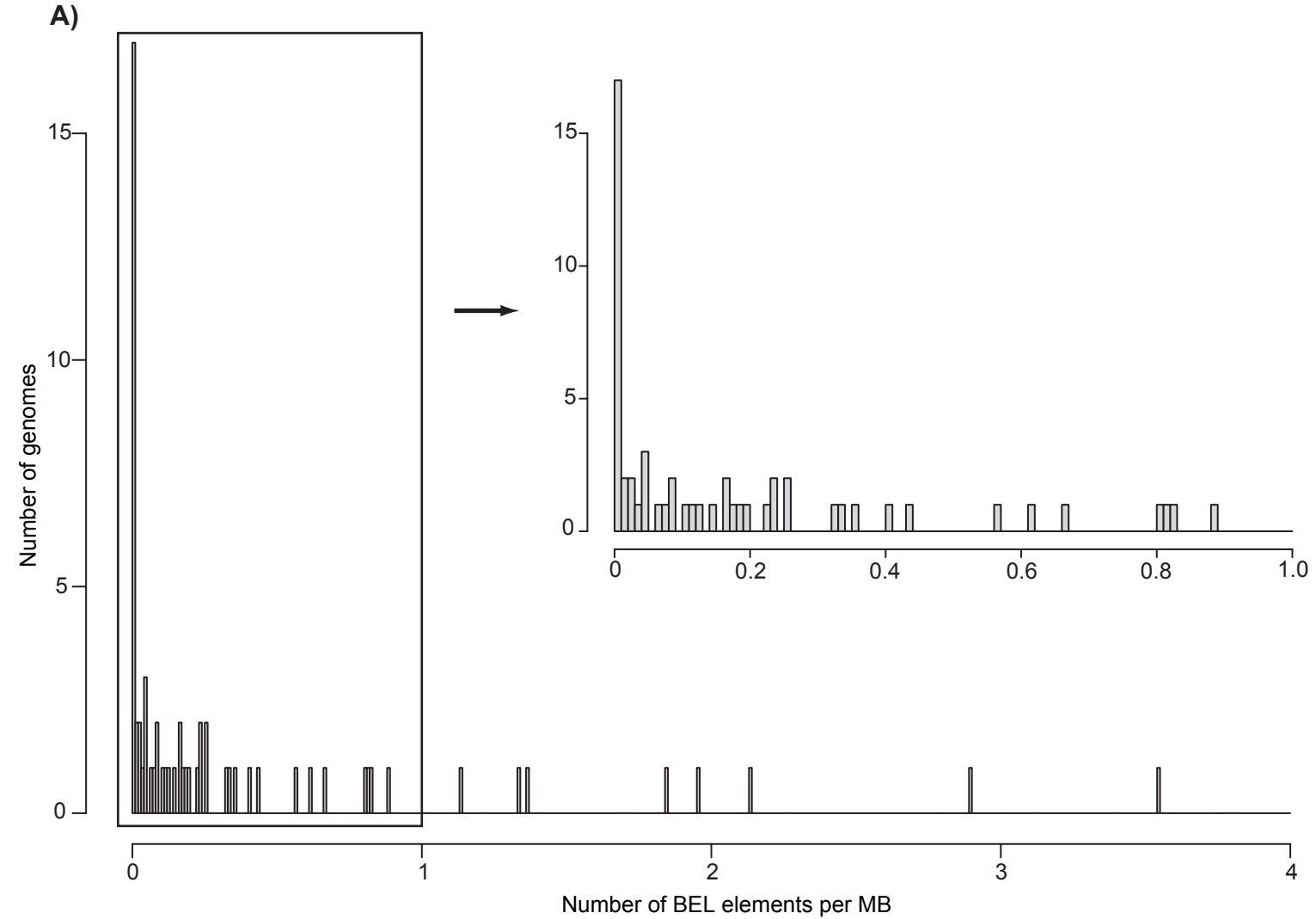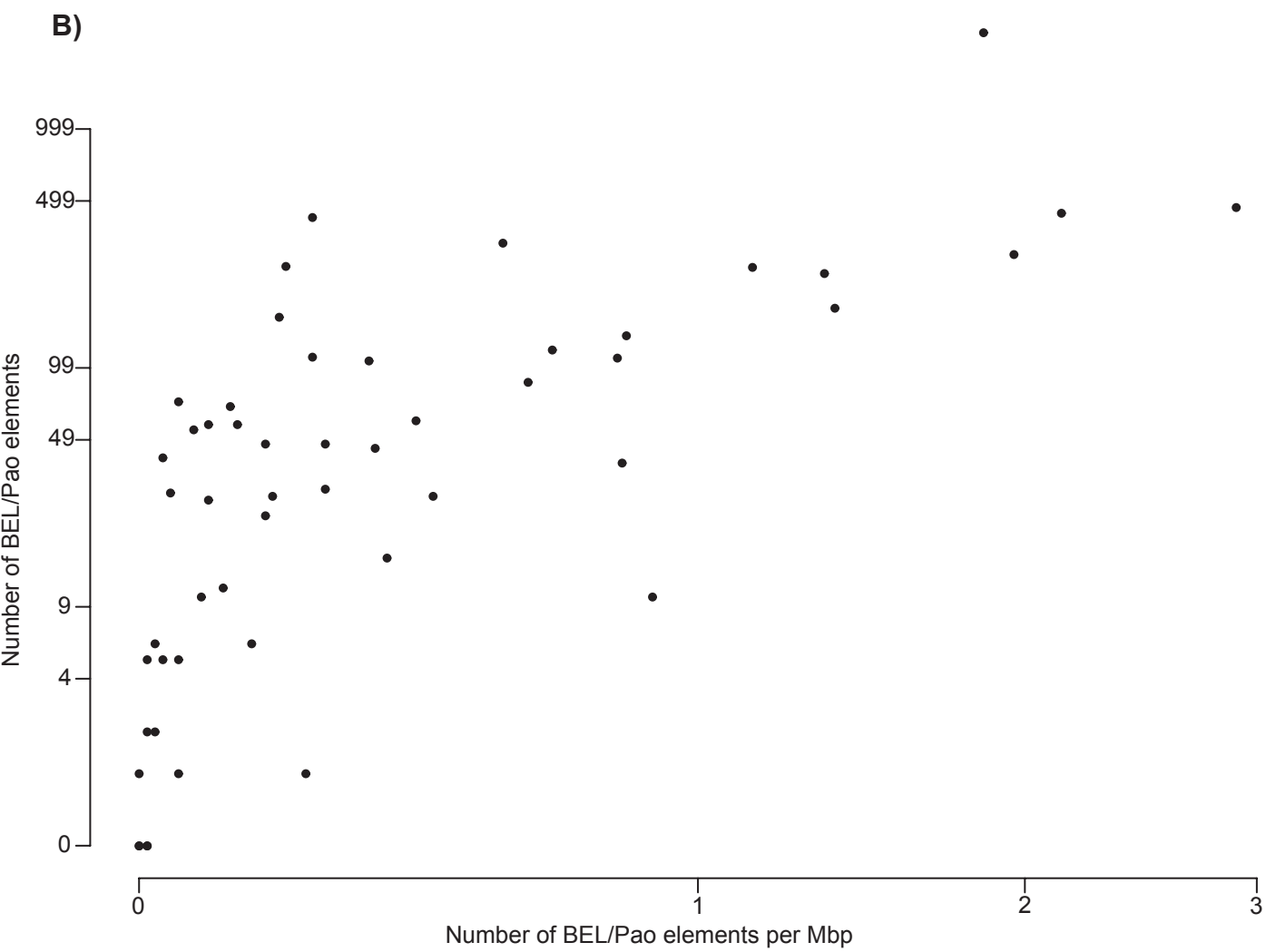

Supplement: Additional file 3 — BEL/Pao copy number per Mbps. A) The histogram shows the number of genomes containing a given copy number of BEL/Pao elements per Mbps. The inset shows the number of genomes containing between zero and one BEL/Pao elements per Mbps. The eight genomes containing more than one BEL/Pao element per Mbps come from either fruit fly or mosquito species. B) Relationship between total copy number and copy number per Mb for each genome. Each point in the graph represents one genome and shows the total BEL/Pao copy number and the copy number per Mbps. Note the logarithmic scale on both axes. [file 1471-2148-11-154-S3.PDF]

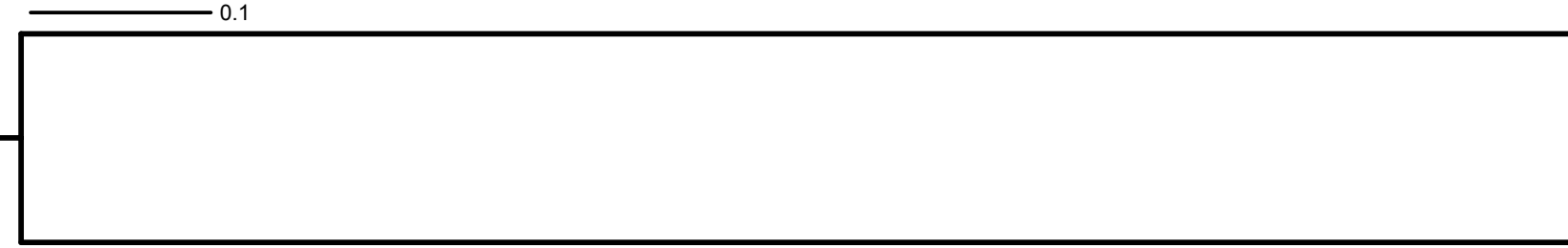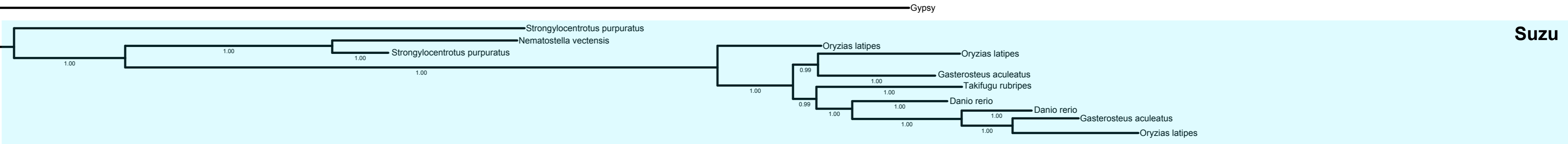

Suzu

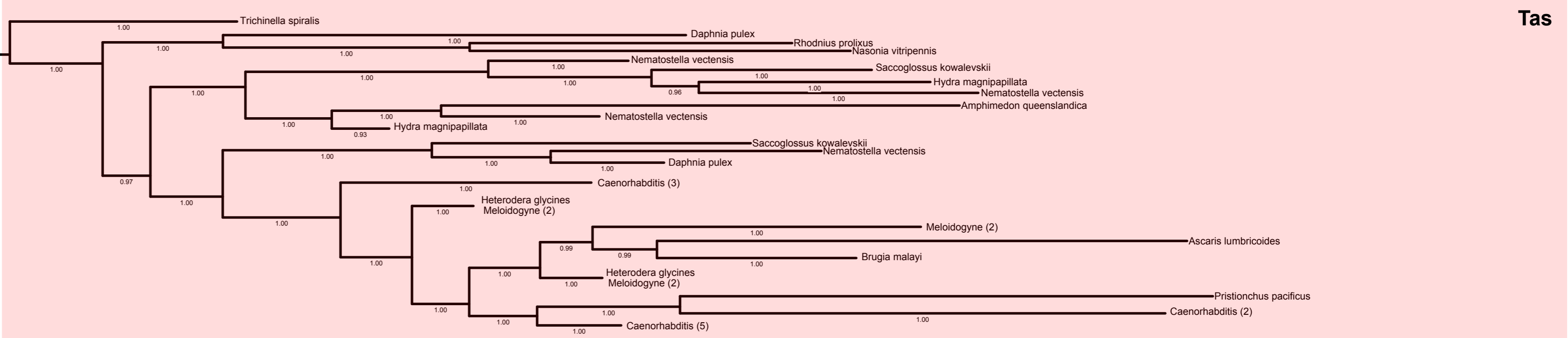

Tas

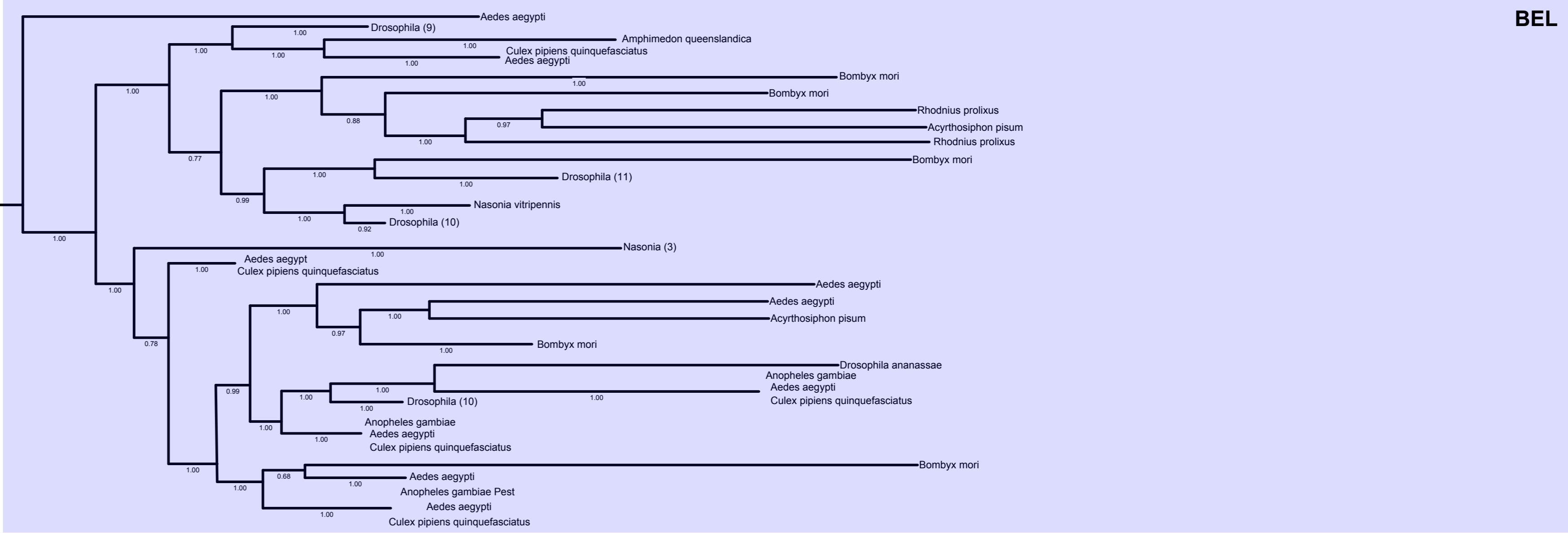

BEL

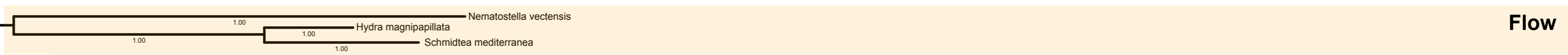

Flow

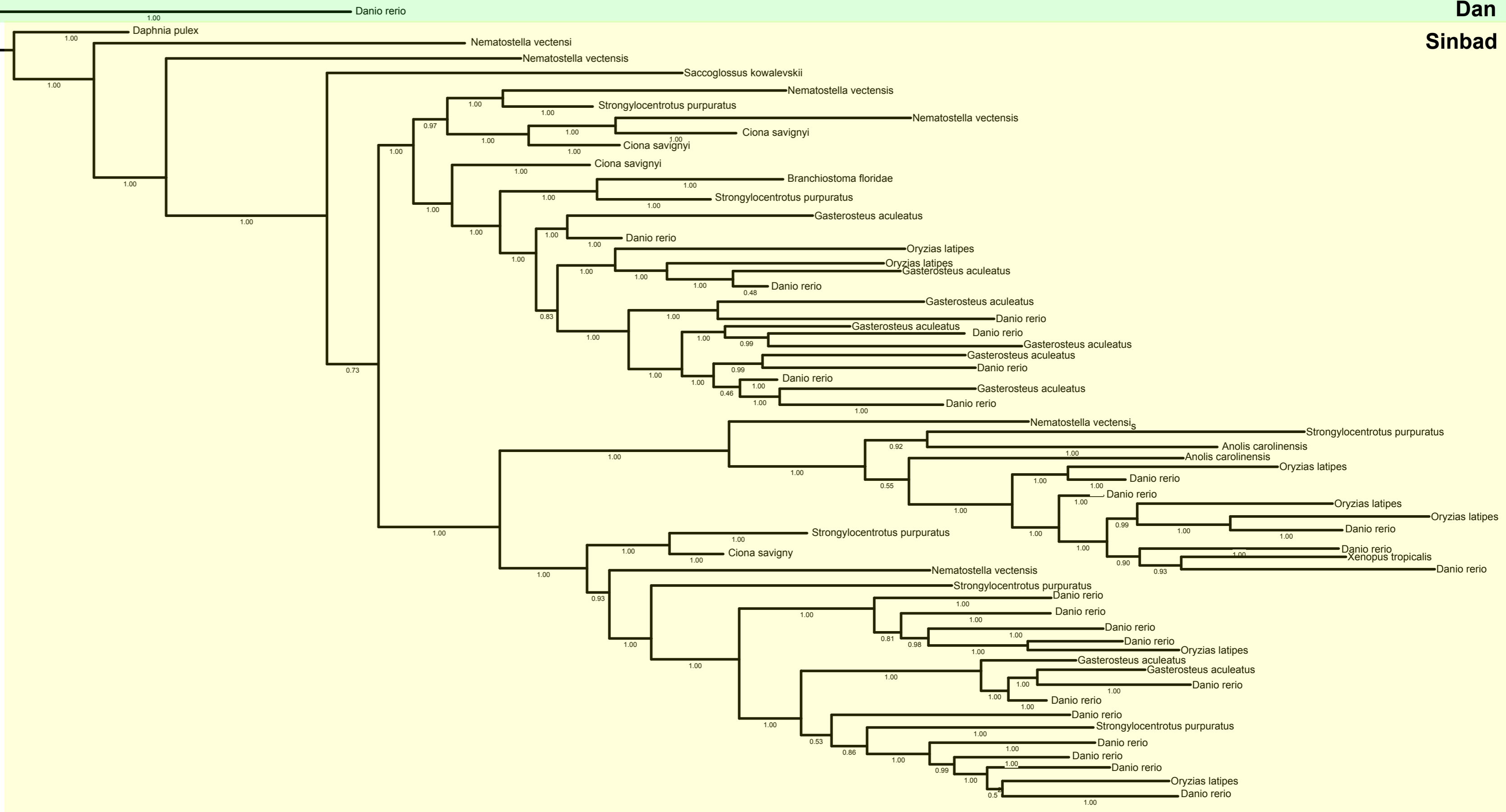

Dan

Sinbad

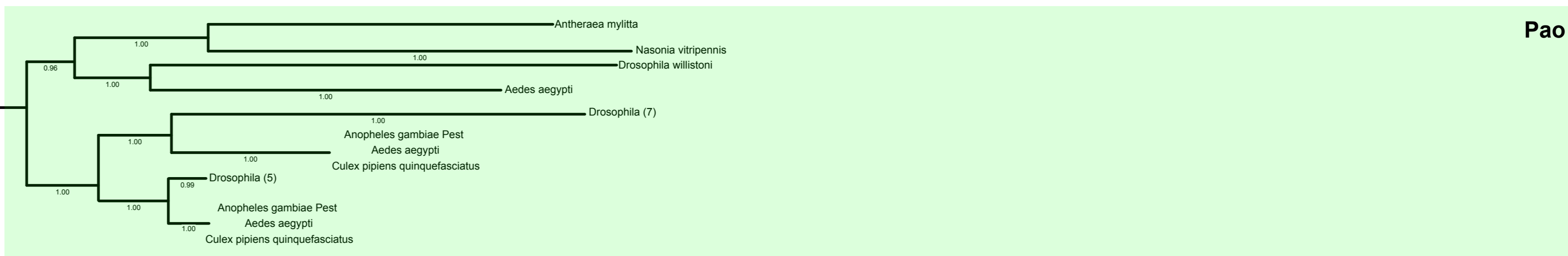

Pao

Supplement: Additional file 8 — Phylogenetic tree of BEL/Pao elements with species names. The Figure shows the same tree as in Figure 4 in the main text but with the species names shown in which the elements occur. If a clade of elements contained only element families from the same species or from very closely related species (e. g. mosquito species), the clade was collapsed to reduce the size of the tree. All species names are shown at the leaves of the tree. If all species in one clade of the tree belonged to the same genus, such as the genus Drosophila, only the genus name is shown, with the number of species in brackets. Major clades are highlighted in different colors. [file 1471-2148-11-154-S8.PDF]
