# Supplementary material for: BEL/Pao retrotransposons in metazoan genomes
Source: BMC Evol Biol. 2011 Jun 4;11:154. doi: 10.1186/1471-2148-11-154 (PMC3118150; doi:10.1186/1471-2148-11-154)
Supplement: Additional file 5 — Species specific family classification. We describe the differences and agreements between the among-species family classification as used in the main text and the within-species family classification. [file 1471-2148-11-154-S5.PDF]

Family classification for each species

In addition to our among-species clustering of families described in the main text, we also clustered the elements we had identified for each species separately ("within-species clustering"). This led to a clustering of our 7,861 BEL/Pao elements into 1050 families with at least two elements and 707 singletons. Most families (922) have a copy number of ten or fewer elements. As in the among-species clustering, only three families (the same three families) have more than 100 copies. The largest family, present in the carolina anole *Anolis carolinensis*, has 397 elements, which is the same as in the among-species clustering. The number of elements in the second largest family, present in *Drosophila ananassae*, increased slightly from 207 elements in the species-wide classification to 214 in the within-species classification. The third most abundant family is present in *D. melanogaster* and contains 111 elements, 32 elements fewer than in the among-species classification. All 32 "missing" elements are from other *Drosophila* species. Overall, the differences between the among-species and within-species classification are small, and regard only the small percentage of families (42 of 1725 families, 2.4 percent) whose members occur in multiple genomes.

The different graphs we built for the BEL/Pao elements of each species during our species specific element clustering, may differ substantially in the connectivity between their nodes. For example, if the elements in a genome are highly similar to one another, then we might expect the corresponding graph to form one large connected component, whereas the graph might be highly fragmented if the elements are dissimilar. Supplemental Figure 2 shows two extreme examples. Supplemental Figure 2A represents the elements in the mosquito *Anopheles gambiae* in a graph that shows many disconnected components, which reflects the overall low sequence similarity of *A. gambiae* elements, and their grouping into clearly distinct families. The elements of the fruit fly *Drosophila persimilis* graph show a completely different relationship (Supplemental Figure 2B). Here, all elements form one big graph, reflecting their generally high similarity. In this case, the algorithm we use can cluster the elements into different families, as shown by the different node colors, but the families are not as well separated as in the example of Supplemental Figure 2A.

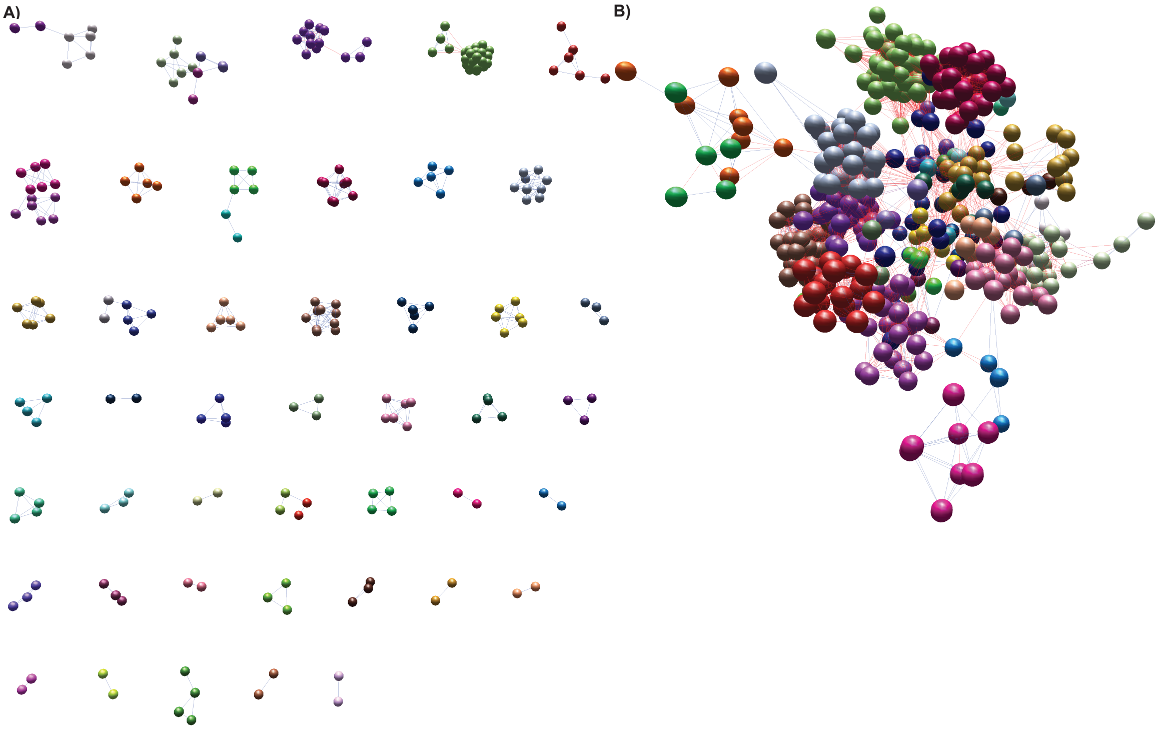

Figure 1: Supplemental Figure 2: Two extreme examples of MCL clustering results. We clustered the BEL/Pao elements from *A. gambiae* (A)) and *D. persimilis* (B)) into families using the MCL algorithm [1] from within BioLayout [2] (see Methods for details). We used default values for the *A. gambiae* data, as described in the Method section. For *D. persimilis* we increased the overlap threshold to 1000 bp, otherwise the graph would show an even higher connectivity. Both species have a high number of BEL/Pao elements (263 and 469 elements, respectively) but the graphs shown have very different connectivity. Specifically, the elements from *A. gambiae* form many disconnected subgraphs (22 unconnected singletons are not shown), whereas the elements from *D. persimilis* form one big connected component, with the exception of 22 unconnected singletons (not shown).

References

[1] Stijn van Dongen. *Graph Clustering by Flow Simulation.* PhD thesis, University of Utrecht, May 2000.

[2] Anton J. Enright and Christos A. Ouzounis. BioLayout–an automatic graph layout algorithm for similarity visualization. *Bioinformatics*, 17(9):853–854, Sep 2001.
