# Supplementary material for: BEL/Pao retrotransposons in metazoan genomes
Source: BMC Evol Biol. 2011 Jun 4;11:154. doi: 10.1186/1471-2148-11-154 (PMC3118150; doi:10.1186/1471-2148-11-154)
Supplement: Additional file 9 — Structural information of superfamilies. The table shows the minimal, maximal, and median element lengths (in basepairs) and the minimal, maximal and median LTR length for the major superfamilies we identified. [file 1471-2148-11-154-S9.PDF]

	min	max	median	min LTR	max LTR	median LTR
BEL	2,338	21,266	7,328	100	2,273	334
Dan	5,431	22,762	7,589	162	2,427	728
Flow	3,303	16,272	6,197	100	812	212
Pao	2,549	21,937	7,478	100	2,210	479
Sinbad	2,170	21,008	7,253	102	2,110	657
Suzu	5,609	21,441	8,139	109	2,419	754
Tas	2,292	21,611	9,421	100	1,814	363
all	2,170	22,762	7,382	100	2,427	383
