# Supplementary material for: BEL/Pao retrotransposons in metazoan genomes
Source: BMC Evol Biol. 2011 Jun 4;11:154. doi: 10.1186/1471-2148-11-154 (PMC3118150; doi:10.1186/1471-2148-11-154)
Supplement: Additional file 10 — List of phyla covered by each superfamily. The table shows the phyla in which BEL/Pao superfamily members were identified. [file 1471-2148-11-154-S10.PDF]

					Eumetazoa				Parazoa	
Phylum	Cnidaria	Platyhelminthes	Mollusca	Nematoda	Arthropoda	Chordata	Echinodermata	Hemichordata	Porifera	Placozoa
BEL	-	-	-	-	+	-	-	-	+	-
Dan	-	-	-	-	-	+	-	-	-	-
Flow	+	+	-	-	-	-	-	-	-	-
Pao	-	-	-	-	+	-	-	-	-	-
Sinbad	+	-	-	-	$(+)^{RU}$	+	+	$(+)^{GyDB}$	-	-
Suzu	+	-	-	-	-	+	+	-	-	-
Tas	+	-	-	+	+	-	-	+	+	-

Table 1: RU: Repbase Update, GyDB: *Gypsy* Database
